# The Theory and Fundamentals of Bioimpedance Analysis in Clinical Status Monitoring and Diagnosis of Diseases

**DOI:** 10.3390/s140610895

**Published:** 2014-06-19

**Authors:** Sami F. Khalil, Mas S. Mohktar, Fatimah Ibrahim

**Affiliations:** 1 Department of Biomedical Engineering, Faculty of Engineering, University of Malaya, 50603 Kuala Lumpur, Malaysia; E-Mails: samifathi@siswa.um.edu.my (S.F.K.); mas_dayana@um.edu.my (M.S.M.); 2 Centre for Innovation in Medical Engineering (CIME), Faculty of Engineering, University of Malaya, 50603 Kuala Lumpur, Malaysia; 3 Department of Biomedical Engineering, College of Engineering, Sudan University of Science and Technology, 407, Khartoum, Sudan

**Keywords:** bioimpedance analysis, body composition, clinical status monitoring, diseases diagnostic and prediction

## Abstract

Bioimpedance analysis is a noninvasive, low cost and a commonly used approach for body composition measurements and assessment of clinical condition. There are a variety of methods applied for interpretation of measured bioimpedance data and a wide range of utilizations of bioimpedance in body composition estimation and evaluation of clinical status. This paper reviews the main concepts of bioimpedance measurement techniques including the frequency based, the allocation based, bioimpedance vector analysis and the real time bioimpedance analysis systems. Commonly used prediction equations for body composition assessment and influence of anthropometric measurements, gender, ethnic groups, postures, measurements protocols and electrode artifacts in estimated values are also discussed. In addition, this paper also contributes to the deliberations of bioimpedance analysis assessment of abnormal loss in lean body mass and unbalanced shift in body fluids and to the summary of diagnostic usage in different kinds of conditions such as cardiac, pulmonary, renal, and neural and infection diseases.

## 1. Introduction

Bioimpedance analysis is a broadly applied approach used in body composition measurements and healthcare assessment systems. The essential fundamentals of bioimpedance measurement in the human body and a variety of methods are used to interpret the obtained information. In addition there is a wide spectrum of utilization of bioimpedance in healthcare facilities such as disease prognosis and monitoring of body vital status. Thus, with such a broad utilization, we feel that this warrants a review of the most fundamental aspects and healthcare applications of bioimpedance analysis.

Studies on the electrical properties of biological tissues have been going on since the late 18th century [1]. Thomasset [2] explored the utilization of bioimpedance measurement in total body water estimation using needle electrodes. Nyboer [3] applied quad surface electrode readings for bioimpedance measurements to estimate the fat free mass of the human body. Hoffer [4] introduced the association between total body impedance and total body water content in reference to tritium dilution techniques.

The electrical properties of biological tissues are currently categorized based on the source of the electricity, *i.e.*, active and passive response. Active response (bioelectricity) occurs when biological tissue provokes electricity from ionic activities inside cells, as in electrocardiograph (ECG) signals from the heart and electroencephalograph (EEG) signals from the brain. Passive response occurs when biological tissues are simulated through an external electrical current source [5]. Bioimpedance or biological impedance is defined as the ability of biological tissue to impede electric current [6].

Due to the noninvasiveness, the low cost and the portability of bioimpedance analysis systems, numerous researchers have conducted studies on bioimpedance analysis and its applications in body composition estimation and evaluation of clinical conditions. Recently, Mialich *et al.* [7] reviewed the applications of bioimpedance analysis in body composition assessment and monitoring of chronic diseases with a comprehensive listing of the most used equations, however, recent techniques such as real time multi-sine bioimpedance analysis and bioimpedance vector analysis methods were not discussed. Lukaski [8] has revised the conceptual modules of bioimpedance analysis for physiological activities assessment and diseases prognosis. The study states that the applied multiple regression approaches and physical modules in bioimpedance analysis have limited utilization in individuals' measurement. This paper is a review of the basic fundamentals and the applications of bioimpedance analysis. The first section highlights the main bioimpedance measurement approaches using single frequency, multiple frequencies and broadband frequency spectrum signals, in addition to applied bioimpedance measurements method across the whole body, through body segments and other alternative analysis method such as vector bioimpedance analysis and real time bioimpedance methods. Body composition parameters, which include lean mass and fluid volumes estimation using bioimpedance measurements, are discussed in the second section. Basic factors in bioimpedance measurements, including anthropometric measurements, age, race, protocols and postures, and shape and artifacts of electrode are discussed in the third section. Finally, applications of bioimpedance analysis in diseases prognosis and clinical monitoring systems are outlined in the fourth section.

## 2. Fundamentals of Bioimpedance Measurement Techniques

Impedance (Z), from an electrical point of view, is the obstruction to the flow of an alternating current and, hence, is dependent on the frequency of the applied current, defined in impedance magnitude (|Z|) and phase angle (φ) as shown in Equations (1)–(3) [9]. Bioimpedance is a complex quantity composed of resistance (R) which is caused by total body water and reactance (X_c_) that is caused by the capacitance of the cell membrane [5]:

(1) $$Z=R+jX_{c}$$

(2) $$\left|Z\right|=\sqrt{R^{2}+X_{c}^{2}}$$

(3) $$\Phi =\tan^{-1}\left(\frac{X_{c}}{R}\right)$$

Resistance of an object is determined by a shape, that is described as length (L) and surface area (A), and material type, that is described by resistivity (ρ), as shown in Equation (4), [9]. Reactance (X_c_) of an object as shown in Equation (5), is defined as resistance to voltage variation across the object and is inversely related with signal frequency (f) and capacitance (C) [9]. In biological systems resistance is caused by total water across the body, and reactance occurs due to the capacitance of the cell membrane [5,10]:

(4) $$R_{\left(\text{ohm}\right)}=\rho_{\left(\Omega .m\right)}\frac{L_{\left(m\right)}}{A_{\left(m^{2}\right)}}$$

(5) $$X_{c(\text{ohm})}=\frac{1}{2\pi f_{\left(Hz\right)}C_{\left(\text{Farad}\right)}}$$

Capacitance (C) is defined as the ability of the non-conducting object to save electrical charges, that is equal to the ratio between differentiation in voltage across object (dV/dt) and current that is passed through the object (I(t)), as shown in Equation (7). In the parallel capacitor module, capacitance is in direct proportion to the surface area (A) in meters square and inversely proportional to distance (d) in meters between the charged plates, and is dependent on the permittivity constant of vacuum (ε_0_ ≈ 8.854 × 10^−12^ F·m^−1^) and the relative dielectric permittivity constant (ε_r_) that is defined based on the material between the plates (for a vacuum space, ε_r_ = 1), as shown in Equation (6) [9]:

(6) $$C_{\left(\text{Farad}\right)}={ɛ}_{0}{ɛ}_{r}\frac{A_{\left(m^{3}\right)}}{d_{\left(m\right)}}$$

(7) $$C_{\left(\text{Farad}\right)}=\frac{dV(t)}{dt}/I\left(t\right)$$

Body composition estimation using bioimpedance measurements is based on determination of body volume (V_b_) through the basic means of resistance measurement. From Equation (4) that gives the relation between resistance and ratio of length (L) to surface area (A), body volume (V_b_) can be obtained by substituting the surface area (A) with the numerator and denominator of the length (L), as in Equation (8):

(8) $$V_{b}\left(m^{3}\right)=\rho_{\left(\Omega .m\right)}\frac{{L^{2}}_{\left(m\right)}}{R_{\left(\text{ohm}\right)}}$$

The human body as a volume is composed generally of fat mass (FM) which is considered as a non-conductor of electric charge and is equal to the difference between body weight (Wt_Body_) and fat free mass (FFM), as shown in Equation (9); and FFM, which is considered as the conducting volume that helps the passing of electric current due to conductivity of electrolytes dissolved in body water. Studies show that water, known as total body water (TBW) is the major compound of FFM and is equal to 73.2% in normal hydration subjects, as in Equation (10) [11]:

(9) $$\text{FM}=Wt_{\text{Body}}-\text{FFM}$$

(10) TBW=0.73FFM

**Figure 1.**
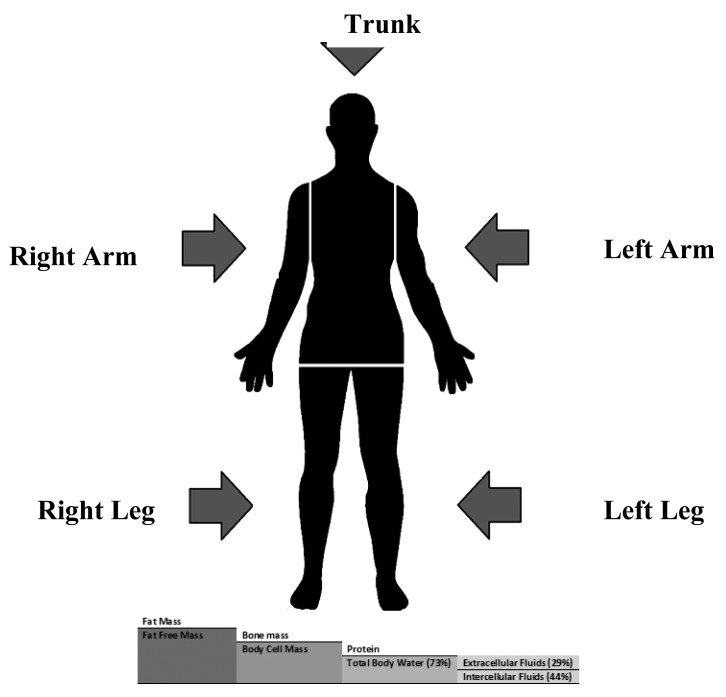
Main body segments and compartments.

In bioimpedance measurements, the human body is divided into five inhomogeneous segments, two for upper limbs, two for lower limbs and one for the trunk. In the five compartment module, the human body is composed of FM and FFM which consists of bone minerals and body cell mass (BCM) that include protein and total body water that consists of extracellular fluid (ECF) and intracellular fluid (ICF) [5]. Figure 1, shows the five segments and compartments of human body.

Most of the known prediction methods rely on the relation between water volume and the ratio between square length to resistance (L^2^/R) [12], however the alternation in anatomical and anthropometric features of the whole human body and segments cause variations in estimated volumes. Jaffrin and Morel reviewed that most TBW estimation equations between 1985 and 1994 were based on values predicted using the H^2^/R_50_ that was introduced by Kyle *et al.* [13,14] and Houtkouper *et al.* [15].

Measurement of bioimpedance is obtained from the whole body and body segments separately, using single frequency, multiple frequencies and bioimpedance spectroscopy analysis. In addition to several alternative assessments method such as bioimpedance vector analysis and real time bioimpedance analysis.

### 2.1. Single Frequency Bioimpedance Analysis (SF-BIA)

Analysis of bioimpedance information obtained at 50 KHz electric current is known as single-frequency bioimpedance analysis (SF-BIA). SF-BIA is the most used and is one of the earliest proposed methods for the estimation of body compartments, It is based on the inverse proportion between assessed impedance and TBW, that represents the conductive path of the electric current [5,16].

SF-BIA predicts the volume of TBW that is composed of fluctuating percentages of extra cellular fluid (ECF) which is almost equal to 75% of TBW, and ICF that represent the rest [5]. SF-BIA instruments have been used to assess TBW and FFM using the derived Equations (2) and (3), respectively, for normal hydrated subjects, although SF-BIA is not valid for body conditions with significantly altered hydration [17]. Studies by Hanai [18] on mixture theory report that body tissue conductivity is diverse [5], and SF-BIA shows limitations in ICF variance prediction, however many of studies show an acceptable correlation in ICF estimation [19].

### 2.2. Multiple Frequency Bioimpedance Analysis (MF-BIA)

Analysis of bioimpedance that is obtained at more than two frequencies is known as multiple-frequency bioimpedance analysis (MF-BIA). MF-BIA is based on the finding that the ECF and TBW can be assessed by exposing it to low and high frequency electric currents, respectively. Thomasset [2] has proposed TBW and ECF estimation using 100 and 1 kHz based on the Cole model [20]. However, in later years, Jaffrin *et al.* [21] stated that technically a bioimpedance analyzer should use frequency range between 5–1000 kHz. Simpson *et al.* [22] state that low frequency in MF-BIA is generally less than 20 KHz and high frequency is more than 50 KHz. Hannan *et al.* [23] report that parameters measured using a frequency of less than 5 KHz and more than 200 KHz fluctuate around the actual value and conclude that estimated TBW is more accurate using the MF-BIA than the BIS method with the same predicted values of ECF for both methods. Patel *et al.* [24] reported that in diseased subjects, TBW prediction using SF-BIA gave more precise results than MF-BIA. In general, the MF-BIA method predicts ECF more precisely than the SF-BIA method; however in elderly diseased subjects the MF-BIA method shows less sensitivity in detecting fluid shifts between ECF and ICF [19].

### 2.3. Bioimpedance Spectroscopy (BIS)

**Figure 2.**
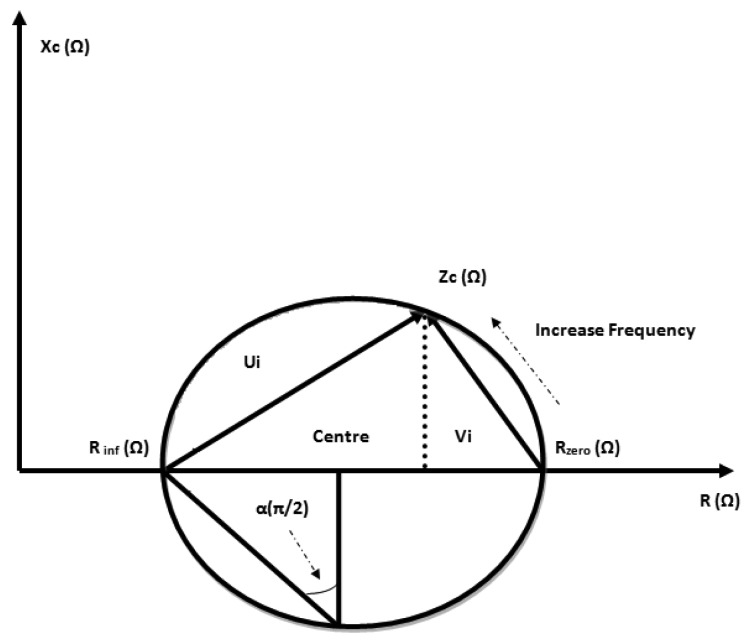
Cole-Cole module plot and Cole module parameters.

Analysis of bioimpedance data obtained using a broad band of frequencies is known as bioimpedance spectroscopy (BIS). The BIS method is based on the determination of resistance at zero frequency (R_0_) and resistance at infinity frequency (R_inf_) that is then used to predict ECF and TBW, respectively. The use of 100 and 1 kHz, respectively, was earlier proposed by Thomasset [25] who applied the basics of Hanai's mixture theory [18] and Cole's module [26,27] as explained by the Cole-Cole plot (Figure 2), however it is complicated to directly measure these values because of the relaxation phenomena of living tissue [20].

Reference methods for estimating TBW are based on radioisotopic dilution of deuterium, and for ECF estimation they are based on the dilution of bromide [28] and for ICF they are based on the radioactive potassium isotope, ^40^K, both elements which are readily diffused in the human body [29,30]. Reference techniques are invasive, expensive and complicated when compared to bioimpedance methods, although the precision is dependent on the electrical module and body parameter variation [21].

Estimation of TBW, ECF and ICF using BIS techniques can be performed using either an equation modules approach [10,31–33] or an analytically derived equations approach [27]. Hanai's mixture theory shows limitations in some studies [14,15,34], however it showed advantages in other studies [35,36]. Ward *et al.* [37] stated that the differences in biological construction among subjects may limit mixture theory as noted in some studies [38,39]. Scharfetter *et al.* [40] report that an accurate module for body fluid allocation and trusted fitting methods are most crucial factors in the BIS method.

The determination of Cole module parameters (R_0_, R_inf_, α, F_c_), in Figure 2 is done using the BIS method which is based on the argument that the human body is composed of a mixture containing conducting and non-conducting compartments [18].

In Equation (4), the reference method is based on the assumption that the measured resistance (R) represents the total conducting volume of the lean body mass. However in the BIS method, the measured resistance represents the total conducting and non-conducting part of the lean body mass, so that the non-conducting part is included by multiplying the obtained resistance by body shape factor (K_b_) and substituting the surface area (A) by body volume (V_b_). Ayllon *et al.* [41] reports that the estimation of Cole module parameters (R_0_, R_inf_, α, F_c_) that is obtained by using only resistance achieves slightly better results and there is less standard error based on the Non-Linear Least Squares technique as compared to the capacitive and impedance complex components. Ward *et al.* [42] concludes that the Cole parameters can be obtained by using four selected frequencies and substituting a fitting technique based on amplitude impedance values at these frequencies:

(11) $$R=K_{b}\rho \frac{Ht^{2}}{V_{b}}$$

where, R is resistance, ρ is resistivity, Ht is the human height, V_b_ is the body volume and K_b_ is a dimensionless shape factor calculated from the length and perimeters of the upper and lower limbs, and the trunk, taken into consideration the body shape composed of the five cylinders.; Van Loan *et al.* [43] calculated the shape factor (K_b_) from statistical anatomical measurements in adults to be equal to 4.3.

### 2.4. Whole Body Bioimpedance Measurement

**Figure 3.**
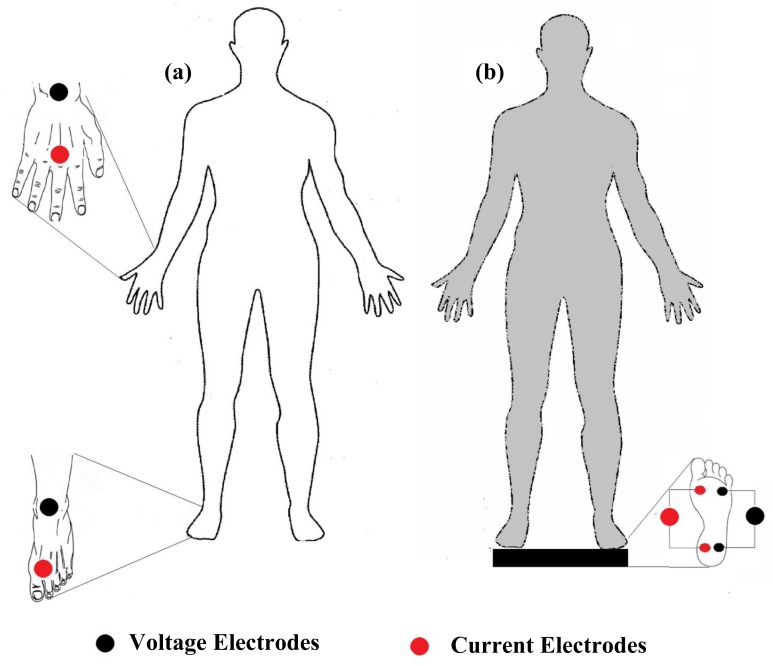
Whole body bioimpedance measurement techniques, (**a**) hand to foot and (**b**) foot to foot electrodes positioning.

Measurement of total body bioimpedance is the most commonly used method for estimating whole body compartments. Many of the whole body bioimpedance instruments apply three approaches for impedance measurement: hand to foot method [14,17], foot to foot [44–46] method and hand to hand method [47,48]. The hand to foot (Figure 3a) one is the most commonly used method. It was introduced by Hoofer [4] and later revised by Nyboer [3] to decrease the contact impedance between skin and electrodes, and validated by Lukaski [17] in 140 normal adults. Tetrapolar hand to foot measurements are performed on a supine subject for 15 min, placing electrodes filled with gel to minimize gap impedance on the dorsal surfaces of the right hand and foot, distal (current) ones being respectively proximal to the metacarpal and metatarsal phalangeal joints, in accordance with standard tetrapolar electrode placement [49]. Foot to foot measurements (Figure 3b) were introduced by Nuñez *et al.* [50] through the use of a pressure-contact foot-pad electrode. In leg to leg bioimpedance measurements, the subject stands vertically, with uncovered feet, on four stainless steel footpads electrodes and divided for each foot into frontal and back portion for current injecting and voltage measurement [46]. Hand to hand bioimpedance measurements were introduced by Ghosh *et al.* [48] by performing body composition analyses using a handheld impedance meter in subjects with malnutrition. The device was held while both arms were stretched out horizontally in front of the body. Deurenberg *et al.* [47] validated the hand to hand method on 298 Singaporean subjects and reported that readings obtained using a handheld impedance meter were significantly acceptable for those subjects.

### 2.5. Body Segment Bioimpedance Measurement

Segmental bioimpedance analysis achieves better estimation of skeletal muscle mass (SMM) than whole body bioimpedance analysis, with a reported standard error of 6.1% in reference to MRI measurements among 30 male subjects [51]. Baumgartner *et al.* [52] stated that multi-frequency segmental bioimpedance analysis enhances and elucidates the relationship between bioimpedance analysis and body compartment estimation after examining the impact of phase angle on body composition prediction among 116 normal subjects.

Segmental bioimpedance analysis detects the fluctuation in ECF due to differences in posture and is more precise than the ankle foot method [53], and gives a better estimation of TBW than total body measurements with reference to dilution method [54].

Segmental or perpendicular bioimpedance analysis defines the measurement method of body segments that is mostly treated as five cylinders as in Figure 1 [5], and was introduced to overcome the disagreement between trunk resistance to upper limbs ratio and trunk resistance to lower limbs ratio of 0.72 and 0.66 respectively [52]; Earthman *et al.* stated that the trunk represents 50% of the body mass [55]. Kyle *et al.* pointed out that total bioimpedance measurement assesses mainly the upper and lower limb compartments, and shows some limitation to predict water compartments of the trunk [13].

**Figure 4.**
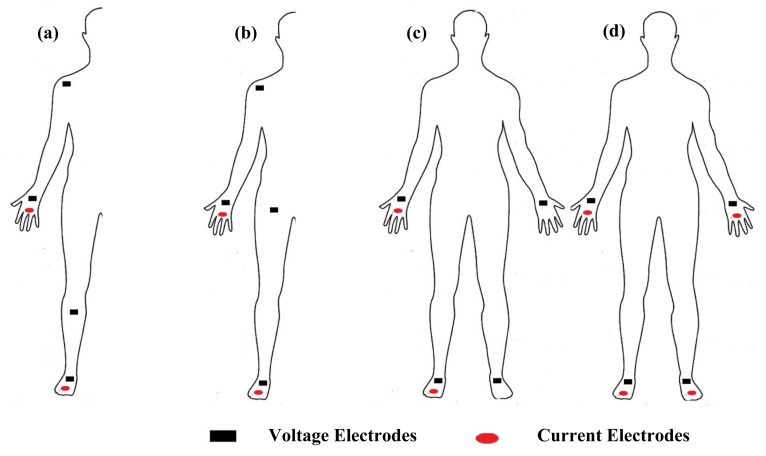
Segmental bioimpedance analysis techniques, (**a**) right side dual current and quad voltage electrodes, (**b**) right side dual current and quad voltage electrodes, (**c**) double sides dual current and quad voltage electrodes and (**d**) double sides quad current and quad voltage electrodes.

Measurement of segmental bioimpedance can be achieved through four types of protocols. The first approach, as suggested by Scheltinga *et al.* [56], uses dual current injection electrodes on the proximal area of the right forearm and lower leg, and quad voltage electrodes placed on the right proximal forearm, shoulder, upper thigh and lower leg (Figure 4a). The second approach is suggested by Zhu *et al.* [57], through the sum of segments technique, that uses dual current injection electrodes on the right wrist and foot, and quad voltage electrodes placed on the right wrist, shoulder, upper iliac spine and foot (Figure 4b). A third approach was presented by Organ *et al.* [58], who suggested the use of dual current injection electrodes on the right wrist and foot, and quad voltage electrodes, two placed on the right wrist and foot, and two on the left wrist and foot (Figure 4c). The fourth approach as suggested by Jaffrin *et al.* [16,59,60], is through the use of quad current injection electrodes located on the right and left wrist and foot, and quad voltage electrodes located at the same place (Figure 4d).

Limitations of whole body bioimpedance measurement in evaluating body segment compartments have given rise to the demand for segment localized bioimpedance analysis applications. Scharfetter *et al.* [40], reported that using segmental (across the waist) localized bioimpedance analysis can significantly estimate abdominal fat with a correlation coefficient of R^2^ = 0.99; furthermore Seward *et al.* [61], introduced localized bioimpedance analysis as a trending diagnostic tool for neuromuscular disorders. The study was applied on 25 neuromuscular patients and 45 normal subjects for control.

Studies report that the segmental bioimpedance analysis method shows some limitations in the estimation of FFM [62,63], with estimation power not significantly different from whole body bioimpedance method [44]. However, Kyle *et al.* [13] concluded that enhancement can be achieved through applying the MF-BIA method and further studies on electrode types and allocation.

### 2.6. Alternative Bioimpedance Analysis Method

**Figure 5.**
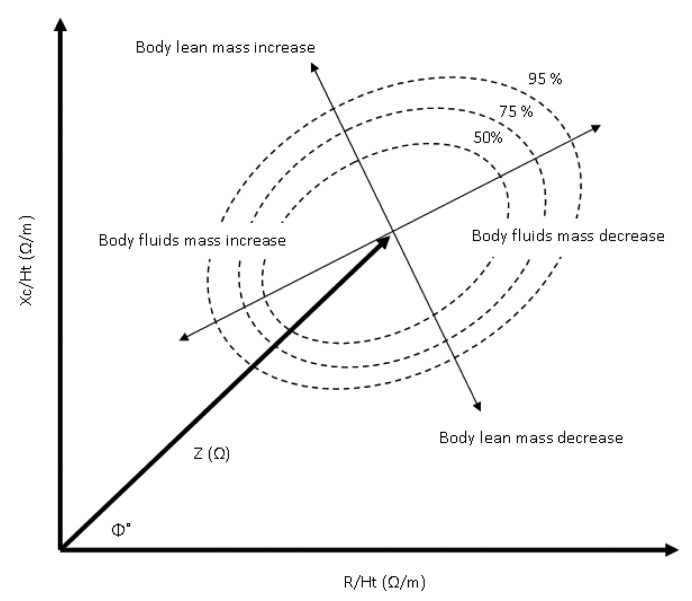
Bioimpedance vector analysis (BIVA) and tolerance ellipses.

Bioimpedance analysis, as an independent method for the assessment of the human health status from absolute bioimpedance measurements, has triggered a new path of data analysis and interpretation. The bioimpedance vector analysis method (BIVA) is a novel approach established essentially by Piccoli *et al.* [64,65] to estimate the hydration status using height indexed resistance and reactance data (R-X_c_ graph) from bioimpedance measurements. Using 8,022 normal subjects (3796 female and 4226 male) Piccoli *et al.* [66] formulated 50%, 75%, and 95% tolerance ellipses that determine increasing and decreasing body mass if the minor vector falls in the left and right half of the 50% ellipse, along with increasing and decreasing hydration ratio if the major vector falls in the lower and upper half of the 50% ellipse (Figure 5).

Evaluation study of the BIVA method by Cox-Reijven *et al.* [67], on 70 diseased subjects with gastrointestinal disorders, conclude the high specificity and low sensitivity of BIVA method in classifying patients with extraordinarily rates of body fluids. Low values (Xc/H < 27.7 O/m and R/H < 563.6 O/m) in the BIVA method can be considered as predictors of severity among diseased children, as shown in a study conducted on 332 precarious pediatric patients with multiple organ dysfunction (MODS), acute respiratory distress syndrome (ARDS) and acute lung injury (ALI) [68].

In [69] the BIVA method successfully monitored rapid increases in ECF during short term recovery (3 weeks) and a dramatic increase in BCM during long term recovery (3 months) among 47% of 57 diseased women with anorexia nervosa [5].

The BIVA method is also considered as a valid tool for the estimation of dry weight in 24 haemodialysis patients' with reference to the Bilbrey Index based on different allocation of values before and after obtrusion [70].

Kyle *et al.* reported that the BIVA method is affected by differences in biological factors and measurement artifacts [5]. Ward and Heithmann state that BIVA is affected by body size and influenced by the cross-sectional area of the body [71].

A specific BIVA method has been proposed by Marini *et al.* [72] to neutralize the bias due to body size. The specific BIVA method used a resistivity-reactivity graph that is constructed using information and results collected from multiplication of resistance and reactance by ratio of cross section area and length (L/A) from Ohm's law (Equation (1)). The cross section area (A) and length (L) were estimated as follows: A = (0.45(arm area) + 0.45(calf area) + 0.10(waist area)) in square meter [73,74], where segment area = c^2^/4π and (c) is the circumference in meter of the arm, waist and calf, respectively; L = 1.1 (Ht), where Ht is body height in meters.

Another alternative method for analysis is real time processing of bioimpedance data which is currently introduced as a key feature for body health monitoring applications. A logarithmic analysis carried out between 0.01 and 10 Hz with five frequencies needs 276 s to be completed, this includes the calculation time [75]. Sanchez *et al.* [76] stated that real time processing, accuracy and the ability of data retrieval and throughput of a BIS system were the most important features to be applied in health monitoring systems, and Sanchez *et al.* [77] introduced a local polynomial based method for impedance-frequency-response estimation. Comparison studies between four different multi-sine periodic broadband excitations broadband for EIS measurements in term of accuracy and speed in frequency and time domain concluded that multi-sine and discrete interval binary sequences (DIBS) enhance SNRZ and have better accuracy than chirp and maximum length binary sequences (MLBS) [75].

Use of multi-sine excitation signals in bioimpedance measurements that is proposed in [78,79] helped increase the accuracy of the measured bioimpedance parameters. It has been validated using a set of optimal multi-sine measurements on 2R-1C equivalent electrical circuits, then applied on healthy myocardium tissue. The multi-sine excitation method was introduced as a parametric-in-time identification method for electrical bioimpedance measurements with inclusion of harmonic impedance spectra (HIS). HIS directly identified from noisy current and voltage myocardium measurements at the multi-sine measurement frequencies to express periodic changes of impedance, rather than the commonly used method that assumed the measurement changing over time [80].

## 3. Body Composition Prediction Using Bioimpedance Analysis

Body composition assessment is considered a key factor for the evaluation of general health status of humans. Several methods use different assumptions to estimate body composition based on the number of compartments. This review considers that the human body is composed of two main compartments, FM and body lean mass or FFM. FFM is composed of bone minerals and body cell mass (BCM) that includes skeletal muscle mass (SMM). BCM contains proteins and TBW that represents 73% of lean mass in normal hydrated subjects. TBW is composed of ICF and ECF as illustrated in Figure 1. In this section, several predictive equations for both lean and fat mass, in addition to body fluids, will be discussed.

### 3.1. Fat Mass (FM) and Fat Free Mass (FFM)

FM and FFM estimations are considered one of the main objectives of body composition assessment techniques. Variations in FM among the reference population are due to several factors, but are believed to follow aging factors in addition to gradual changes in lifestyle [81].

Anthropometric and skin fold thickness measurements are traditional, simple and inexpensive methods for body fat estimation to assess the size of specific subcutaneous fat depots [82] compared with other methods such as underwater weighing, dilution method and dual-energy x-ray absorptiometry [DXA] that requires a trained practitioner to perform it.

Bioimpedance analysis has been shown in recent studies to be more precise for determining lean or fat mass in humans [83]. In comparison with BMI, anthropometric and skin fold methods, BIA offers trustable results in the estimation of fatness across human tissues [84]. Several studies conducted to establish reference values for FFM are based on bioimpedance measurements.

Kyle *et al.* [13] developed a single Equation (12) for the prediction of FFM, using 343 normal subjects aged from 22 to 94 years old, with body mass indexes between 17.0 and 33.8 kg/m^2^ in reference to DXA method:

(12) $$\begin{array}{cc} \text{FFM} & =-4.104+0.518ht^{2}/R_{50}+0.231wt+0.130X_{c,50})+4.229\text{sex}\\ & \text{Sex}\{\begin{array}{c} 1,\text{Male}\\ 0,\text{Female} \end{array} \end{array}$$

where (Ht) is body height, (R_50_) and (X_C, 50_) is resistance and reactance at 50 KHz, and (Wt) is body weight. The developed equation achieved a correlation coefficient (R) that is equal to 0.986, standard error of the estimate (SEE) is equal to 1.72 kg and technical error is 1.74 kg.

In [81,85], FFM was assessed in a population of 5,225 white subjects aged from 15 to 98 years old using bioimpedance measurements and it was concluded that mean FFM was 8.9 kg or 14.8% lower in men older than 85 years than in men 35 to 44 years old and 6.2 kg or 14.3% lower in women older than 85 years than in women 45 to 54 years old.

Sun *et al.* [86], used a multi-component model based on densitometry, isotope dilution, and dual-energy X-ray absorptiometry to build Equations (13) and (14) for FFM estimation:

(13) $${\text{FFM}}_{\text{male}}=-10.68+0.65ht^{2}/R_{50}+0.26wt+0.02R_{50}$$

(14) $${\text{FFM}}_{\text{female}}=-9.53+0.69ht^{2}/R_{50}+0.17wt+0.02R_{50}$$

The mean FFM prediction equations achieved a correlation coefficient *R*^2^ = 0.90 and 0.83 and root mean square errors of 3.9 and 2.9 kg for males and females, respectively.

Deurenberg *et al.* [87], used densitometry, anthropometry and bioelectrical impedance to formulate FFM prediction Equation (15) using 661 normal adult subjects aged from 7 to 83 years old:

(15) $$\begin{array}{cc} \text{FFM} & =-12.44+0.34Ht^{2}/R_{50}+0.1534Ht+0.273Wt-0.127\text{Age}+4.56\text{Sex}\\ & \text{Sex}\{\begin{array}{c} 1,\text{Male}\\ 0,\text{Female} \end{array} \end{array}$$

The FFM prediction equations achieved a correlation coefficient *R*^2^ = 0.93 and standard estimation error (SEE) = 2.63 kg.

Pichard *et al.* [88], assessed FFM and FM in a 3,393 white subject population aged from 15 to 64 years old using bioimpedance measurements and performed a comparison of %FM as determined by BIA with %FM determined by calculations using BMI developed by Deurenberg *et al.* [89], and concluded that the mean FFM ranged of 59.1–61.0 kg for men and 43.3–44.1 kg for women which is 38% greater in men.

Heitmann [90] compared three body composition methods (BMI, skin folds and BIA) using 139 healthy subjects aged from 35 to 65 years old:

(16) $$\text{FM}=14.94-0.079Ht^{2}/R_{50}+0.818wt-0.231ht-0.064\;\text{sex wt}+0.077\text{Age}$$

The multiple regression Equation (16) for impedance had a higher correlation coefficient (R^2^ = 0.89) and lower standard estimation error (SEE = 3.32 kg) than the multiple regression equations for skin fold (R^2^ = 0.81, SEE = 3.91 kg) or body mass index (R^2^ = 0.85, SEE = 3.94 kg).

Heitmann [88] assessed FFM and FM in 2987 out of a 3608 subject Danish population aged from 35 to 65 years old. The obtained data, which are estimated from measurements of electrical impedance, concluded that men have a FM of 4.5 kg, an increase by 30%, when compared to women that have a 6.9 kg increase of 36% for evaluated sample.

Recently, Pichler *et al.* [91] assessed FM in 116 subjects (32 healthy subjects and 84 patients) and concluded that the following prediction equation overestimated FM by 6.55 ± 3.86 kg:

(17) $$FM_{\text{Male}}=-18.42+0.60Wt-0.57\frac{Ht^{2}}{R_{\text{tbw}}}+0.62\frac{Ht^{2}}{R_{\text{ecf}}}$$

(18) $$FM_{\text{Female}}=-9.81+0.65Wt-0.66\frac{Ht^{2}}{R_{\text{tbw}}}+0.65\frac{Ht^{2}}{R_{\text{ecf}}}$$

where R_ecf_ and R_tbw_ represents resistance of extracellular fluids and total body water extracted using the Cole module [26]. In conclusion, all studies state that the men have higher estimated FM as compared to women. Moreover, FFM for both genders decreases progressively with increasing age [81,88].

### 3.2. Body Fluids

Body fluid is the total volume of fluids inside a human body that represents the majority of the FFM volume percentage. TBW includes the fluids inside the cellular mass that is known as ICF; and the fluid located outside the cell body which is composed of plasma and interstitial fluid which is known as ECF. ECF and ICF fluids that are incorporated under TBW, contain several ion types with different concentrations, however the main ions in ECF are Na^+^ and Cl^−^, and for ICF are K^+^ and PO^−4^ [92].

Body fluids estimation using bioimpedance measurements are based on the inversely proportional between body resistance and the total amount of body water [93]. There are varieties of methods for estimating body fluid volumes based on bioimpedance analysis approach.

Sun *et al.* [86] developed prediction Equations (19) and (20) of the TBW reference to dilution method using SF-BIA from a multi-ethnic pool of 1830 people aged from 12 to 94 years old:

(19) $${\text{TBW}}_{\text{male}}=1.2+0.45Ht^{2}/R_{50}+0.18Wt$$

(20) $${\text{TBW}}_{\text{female}}=3.75+0.45Ht^{2}/R_{50}+0.11Wt$$

The developed equation achieved a correlation coefficient (R^2^) and mean square error equal to 0.84 and 3.8 L in men, and 0.79 and 2.6 L in women.

For ECF and ICF estimation using SF-BIA, a few studies performed were based on measurement of bioimpedance in 50 KHz frequency, Sergi *et al.* [94], predict ECF using two frequencies (1 and 50 KHz):

(21) $${\text{ECF}}_{50\mathrm{KHz}}=-5.22+0.20Ht^{2}/R_{50}+0.005Ht^{2}/Xc_{50}+0.08Wt+1.9\text{Health}+1.86\text{Sex}$$

(22) $$\begin{array}{cc} {\text{ECF}}_{50\mathrm{KHz}} & =-7.24+0.34Ht^{2}/R_{1}+0.06Wt+2.63\text{Health}+2.57\text{Sex}\\ & \text{Sex}\{\begin{array}{l} \begin{array}{ll} 1 & ,\text{Male} \end{array}\\ \begin{array}{ll} 0 & ,\text{Female} \end{array} \end{array};\text{Health}\{\begin{array}{l} \begin{array}{cc} 1 & ,\text{Healthy} \end{array}\\ \begin{array}{cc} 2 & ,\text{Diseased} \end{array} \end{array} \end{array}$$

After measurements performed using bioimpedance and bromide dilution methods on 40 subjects (19 males and 21 females) aged 21–81 years, of which 22 were healthy subjects, 12 were affected by chronic heart failure and 6 by chronic renal failure, the best estimation results at 1 KHz achieved a correlation coefficient (R^2^) and standard estimation error equal to 0.89 and 1.7 L.

Due to incomplete conduction of the intracellular fluid at 50 kHz [2], MF-BIA was proposed to increase accuracy of estimation of TBW, ECF and ICF. Deurenberg *et al.* [95] used MF-BIA (1, 5, 50, 100 KHz) to predict TBW using Z_100KHz_ and Z_50KHz_; and ECF using Z_1KHz_ and Z_5KHz_ among 139 normal adult subjects with reference to deuterium oxide dilution and bromide dilution:

(23) $${\text{TBW}}_{100\text{KHz}}=6.69+0.34573Ht^{2}/Z_{100}+0.17065Wt-0.11\text{Age}+2.66\text{Sex}$$

(24) $$\begin{array}{cc} {\text{TBW}}_{50\text{KHz}} & =6.53+0.36740Ht^{2}/Z_{100}+0.17531Wt-0.11\text{Age}+2.83\text{Sex}\\ & \text{sex}\{\begin{array}{cc} 1 & ,\text{Male}\\ 0 & ,\text{Female} \end{array} \end{array}$$

The prediction equation of TBW achieved a correlation coefficient (R^2^) and standard error of estimate (SEE) equal to 0.95 and 1.73 L using Z_100KHz_, and 0.95 and 1.74 L using Z_50KHz_:

(25) $${\text{ECF}}_{1\text{KHz}}=2.30+0.19528Ht^{2}/Z_{1}+0.06987Wt-0.02\text{Age}$$

(26) $${\text{ECF}}_{5\text{KHz}}=2.53+0.18903Ht^{2}/Z_{5}+0.06753Wt-0.02\text{Age}$$

The prediction equation of ECF achieved a correlation coefficient (R^2^) and standard error of estimate (SEE) equal to 0.87 and 0.98 L using Z_1KHz_, and 0.86 and 1.02 L using Z_5KHz_.

Prediction of body fluids using the BIS method in three steps involves firstly determination using the values of R_e_ from R_0_ and R_inf_, secondly, inclusion of the body shape factor K_b_ due to the variation of body segments, and thirdly, inclusion of apparent resistivity ρ_a_ instead of the general resistivity ρ as stated by Hanai in mixture theory [18]:

(27) $$\rho_{a}=\frac{\rho }{{\left(1-c\right)}^{\frac{3}{2}}}$$

where (*c*) is volume fraction of non-conducting tissue. Based on Hanai's mixture method [18], tissue resistance (R) is measured based on conductive tissue, so it should exclude non-conducting tissue. Thus, by substituting Equation (27) in Equation (11), the apparent resistance (R_a_) can be calculated using the following Equation (28):

(28) $$R_{a}=\frac{K_{b}\rho Ht^{2}}{V_{b}{\left(1-c\right)}^{3/2}}$$

At low frequencies the current will pass through extracellular fluids only without intracellular fluid due to the high capacitance of cell membranes [96]. In that case the conducting volume is equal to the ratio between ECF volume (V_ecf_) and TBW volume (V_b_). The volume fraction of non-conducting tissues at low frequencies calculated as in Equation (29):

(29) $$c=1-\frac{V_{\text{ecf}}}{V_{b}}$$

Based on the mixture theory [18], apparent resistivity (ρ_a_) at low frequency represents the extracellular fluid resistivity (ρ_Aecf_), thus the resistance of ECF (R_ecf_) can be recalculated in Equation (31), by substituting Equation (29) in Equation (28) and including the outcome of apparent resistivity (ρ_aecf_) from Equation (30):

(30) $$\rho_{\text{aecf}}=\rho_{\text{ecf}}{\left(\frac{V_{b}}{V_{\text{ecf}}}\right)}^{3/2}$$

(31) $$R_{\text{ecf}}=K_{b}\rho_{\text{ecf}}\frac{{V_{b}}^{1/2}}{{V_{\text{ecf}}}^{3/2}}$$

Hanai [18], calculated ρ_ecf_ to be equal to 40.3 Ω ·cm for men and 42.3 Ω ·cm for women, which is close to that achieved by saline, and is about 40 Ω ·cm t for the ECF composed of plasma and interstitial water [49].

To reform the equation to evaluate the variance in ECF volume (V_ecf_) caused by changes in estimated ECF resistance (R_ecf_), that is achieved by replacing body volume (V_b_), that is equal to the ratio between body weight (*Wt*) *in Kg* and body density (*D_b_)* in Kg/L from Equation (32) in Equation (33):

(32) $$V_{b}=\frac{Wt}{D_{b}}$$

(33) $$R_{\text{ecf}}=K_{b}\rho_{\text{ecf}}\frac{{\left(\frac{Wt}{D_{b}}\right)}^{1/2}}{{V_{\text{ecf}}}^{3/2}}$$

Body factor (K_b_), extracellular fluid resistivity (ρ_aecf_) and body density (*D_b_*) are constant values that can be included in one factor defined as extracellular fluid factor (K_e_) as in Equation (34), and for extracellular fluid volume (V_ecf_) as in Equation (35):

(34) $$K_{e}=10^{-2}{\left(\frac{K_{b}\rho_{\text{ecf}}}{D_{b}^{\frac{1}{2}}}\right)}^{2/3}$$

(35) $$V_{\text{ecf}}=K_{e}{\left(\frac{Ht^{2}{\left(Wt\right)}^{\frac{1}{2}}}{R_{\text{ecf}}}\right)}^{2/3}$$

Van Loan *et al.* [43], calculated K_e_ using the bromide dilution method to be equal 0.306 for men and 0.316 for women; and the ratio between ρ_icf_ and ρ_ecf_ to be equal to 3.82 for men and 3.40 for women. De Lorenzo *et al.* [10] calculated K_e_ to be equal to 0.229 in women; and ρ_ecf_ to be equal to 40.5 Ω ·cm and 39.0 Ω ·cm for men and women, respectively; and the ratio between ρ_icf_ and ρ_ecf_ to be equal to 6.76 for men and 6.79 for women.

Ellis and Wong [30], analyzed the BIS method as introduced by Van Loan *et al.* [43], with reference to the H_2_O and Br dilution technique in 469 multi-ethnic healthy subjects. The study suggested that the ratio between ρ_icf_ and ρ_ecf_ is equal to 3.032 for men and 2.694 for women, due to underestimation of TBW caused by misprediction of ICF measurements. Biasing factors and different regression module approaches caused slight differences in the ratios obtained by these researchers [30].

Moissl *et al.* [97], suggested a body composition spectroscopy method through recalculating K_ecf_ , using different assumptions through inclusion of body mass index (BMI) and taking the module of non-conducting tissue factor (c) in Equation (14) as a valid assumption, as in Equation (36), and then determining the (V_ecf_) using the same equation as Equation (20):

(36) $$K_{\text{ecf}}=\left(\frac{a}{\text{BMI}+b}\right)$$

From [97], (a) and (b) were calculated to be equal to 0.188 and 0.2883 based on measurements using the Br dilution method as a reference method on dialyzed patients and 120 healthy subjects. At high frequencies, the current will pass through the whole TBW which is composed of ECF and ICF [96], so the conducting volume is equal to the ratio between TBW and total body volume.

Jaffrin *et al.* [31] suggested calculating the TBW directly from R_inf_ using the same assumption of mixture theory [96], and assuming uniformity of water compartments inside human body. Thus, using the same assumption as in Equation (29), the volume fraction of non-conducting tissue (c) at high frequencies can be calculated using Equation (37):

(37) $$c=1-\frac{V_{b}}{V_{\text{tbw}}}$$

To determine the apparent resistivity of total body water (ρ_a_tbw_) from actual total body water resistivity (ρ_tbw_), the parameters in (c) from Equation (37), was included into Equation (38):

(38) $$\rho_{\text{a\_tbw}}=\rho_{\text{tbw}}{\left(\frac{V_{b}}{V_{\text{tbw}}}\right)}^{3/2}$$

By replacing the actual resistivity by apparent resistivity for total body water in Equation (11), and restoring the value of (V_b_) from Equation (32), Equation (40) to determine the total body water factor (K_tbw_) and total body water volume (V_tbw_) is recalculated by using Equation (39):

(39) $$K_{\text{tbw}}={\left(\frac{K_{b}\rho_{\text{tbw}}}{{D_{b}}^{1/2}}\right)}^{2/3}$$

(40) $$V_{\text{tbw}}=k_{\text{tbw}}{\left(\frac{Ht^{2}Wt^{\frac{1}{2}}}{R_{\text{tbw}}}\right)}^{2/3}$$

Considering that total body water is equal to the accumulation of ECF and ICF, Jaffrin *et al.* [31] calculated ρ_tbw_ to be equal to 104.3 Ω ·cm in men and 100.5 Ω ·cm. A validation study conducted in 28 dialysed patients [31], concluded that ρ_tbw_ was equal to 108.1 Ω·cm in men and 100.2 Ω·cm, which predicted 91% of mean water loss when compared with 39% for Cole method [43], but overestimated TBW compared to the original BIS method in 21 healthy subjects with the same ρ_tbw_ and hydration rate values.

For ICF prediction using a BIS method, Matthie *et al.* [32] introduced a second generation mixture theory to overcome the limitations of the first generation in predicting intracellular fluid volume (V_icf_) using a new assumption for TBW resistivity (ρ_tbw_), as in Equation (32):

(41) $$\rho_{\text{tbw}}=\rho_{\text{icf}}-\left(\rho_{\text{icf}}-\rho_{\text{ecf}}\right){\left(\frac{R_{\text{tbw}}}{R_{\text{ecf}}}\right)}^{\frac{2}{3}}$$

In the second version of mixture theory, total body water volume is considered to be equal to the summation of ECF and ICF, for ECF estimation, the relation in Equation (35) is considered as a valid method, and for ICF estimation, the method uses Equation (42); note that the ratio (R_tbw_/R_ecf_) is opposite and proportional to (V_tbw_/V_ecf_):

(42) $$V_{\text{icf}}=V_{\text{ecf}}.\left({\left[\frac{\rho_{\text{tbw}}.R_{\text{ecf}}}{\rho_{\text{ecf}}.R_{\text{tbw}}}\right]}^{2/3}-1\right)$$

Moissl *et al.* [97] calculated *ρ*_icf_ to be equal to 273.9 Ω·cm and *ρ*_ecf_ = 40.5 Ω·cm in men and 264.9 Ω·cm and 39.0 Ω·cm, respectively in women. De Lorenzo *et al.* [10] suggest the formula in Equation (34) to determine intracellular volume (V_icf_):

(43) $${\left(1+\frac{V_{\text{icf}}}{V_{\text{ecf}}}\right)}^{5/2}=\frac{R_{\text{icf}}+R_{\text{ecf}}}{R_{\text{icf}}}\left(1+\frac{\rho_{\text{icf}}V_{\text{icf}}}{\rho_{\text{ecf}}V_{\text{ecf}}}\right)$$

Jaffrin and Morel [21] claim that the prediction of ECF by Hanai [18] mixture theory is valid and direct, however the ICF prediction by De Lorenzo *et al.* [10], who state that the determination of *R*_i_ is less accurate than for *R*_e_ in parallel module because it sums up the errors on *R*_e_ and *R*_inf_, is not.

Moissl *et al.* [97] introduced a different method for calculation of intracellular fluid volume (V_icf_), taking into consideration that the non-conducting tissue factor (c) is as given in Equation (44):

(44) $$c=1-\frac{V_{\text{icf}}}{V_{b}}$$

Then the recalculated intracellular fluid factor (K_icf_) and intracellular fluid volume (V_icf_) are added as in Equations (45) and (46), respectively, and it is concluded that total body water factor (K_tbw_) and total body water volume (V_tbw_) is equal to the summation of ECF and ICF volumes as in Equation (47) and recalculated (V_tbw_) using different assumption of (K_tbw_) and (ρ_tbw_) from Jaffrin *et al.* [31], and Matthie *et al.* [32], as given in Equations (48) and (49):

(45) $$K_{\text{icf}}=\left(\frac{c}{\text{BMI}+d}\right)$$

(46) $$V_{\text{icf}}=K_{\text{icf}}{\left(\frac{Ht^{2}Wt^{1/2}}{R_{\text{icf}}}\right)}^{\frac{2}{3}}$$

(47) $$V_{\text{tbw}}=\left(V_{\text{ef}}+V_{\text{if}}\right)={\left(Ht^{2}Wt^{1/2}\right)}^{\frac{1}{3}}\left(\left(\frac{k_{\text{ef}}}{R_{\text{ecf}}^{\frac{2}{3}}}\right)+\left(\frac{k_{\text{if}}}{R_{\text{icf}}^{\frac{2}{3}}}\right)\right)$$

(48) $$K_{\text{tbw}}=K_{\text{ecf}}{\left(\frac{R_{\text{tbw}}}{R_{\text{ecf}}}\right)}^{2/3}+K_{\text{icf}}{\left(\frac{R_{\text{tbw}}}{R_{\text{icf}}}\right)}^{2/3}={\left(\frac{K_{b}\rho_{\text{tbw}}}{{D_{b}}^{1/2}}\right)}^{2/3}$$

(49) $$\rho_{\text{tbw}}=\left(\frac{{D_{b}}^{1/2}}{K_{b}}\right)\left[K_{\text{ecf}}{\left(\frac{R_{\text{tbw}}}{R_{\text{ecf}}}\right)}^{2/3}+K_{\text{icf}}{\left(\frac{R_{\text{tbw}}}{R_{\text{icf}}}\right)}^{2/3}\right]$$

where (c) and (d) are calculated to be equal to 5.8758 and 0.4194 in [97], when using the ^40^K isotope [98] as a reference method on dialyzed patients and 120 healthy subjects.

Fenech and Jaffrin [2] state that ECF prediction using segmental bioimpedance analysis in supine position (0.79 liter) is less than Watson anthropomorphic method [3] (1.12 liter) and for ICF is reduced by 3.4% for segmental bioimpedance and 3.8% for the Watson anthropomorphic method [3]:

(50) $$V_{\text{tbw},\text{Male}}=2.447-0.09156\;\text{Age}+0.1074\;Ht+0.3362\;Wt$$

Pichler *et al.* [91] examined the BIS method using an Impedimed device (SFB7) in TBW, ECF and FFM with reference to the deuterium space method, sodium bromide space method and DXA method, respectively. The study was applied on 32 healthy subjects and 84 patients with different types of diseases (congestive heart failure, coronary heart disease, essential hypertension, atherosclerosis, kidney disease, chronic renal failure, gastrointestinal diseases, type II diabetes, morbid obesity, osteoporosis, cancer, chronic polyarthritis and anorexia nervosa):

(51) $$V_{\text{tbw\_Male}}=8.75+0.23Wt+0.21\frac{Ht^{2}}{R_{\text{tbw}}}$$

(52) $$V_{\text{tbw\_Female}}=5.94+0.19Wt+0.24\frac{Ht^{2}}{R_{\text{tbw}}}$$

(53) $$V_{\text{ecf\_Male}}=0.11+0.11Wt+0.24\frac{Ht^{2}}{R_{\text{ecf}}}$$

(54) $$V_{\text{ecf\_Female}}=1.24+0.09Wt+0.28\frac{Ht^{2}}{R_{\text{ecf}}}$$

Pichler's equations for TBW achieved a correlation coefficient 0.91 and 0.89 for men and women, respectively, as in Equations (51) and (52). For ECF it achieved 0.87 and 0.89 for men and women, respectively, as in Equations (53) and (54) [91]. Hanai mixture equations [18], when applied in SFB7 give ECF measurements higher than the sodium bromide space method by mean ± SD (0.93 ± 2.62 Liter) however it is noted that the Hanai mixture equations applied in SFB7 detect ECF excess in 9 patients, and TBW measurements higher than the deuterium space method by mean ± SD (3.82 ± 3.37 Liter), and FFM measurements lower than the DXA method by mean ± SD (6.55 ± 3.86 kg).

## 4. Bioimpedance Measurement Biasing Factors

### 4.1. Anthropometric Measurements

Anthropometric measurements such as weight, height, skin fold thickness, lengths, diameters and circumferences that involves mathematical modules are the main contributors in the estimation of body compartments [5,99].

Bioimpedance parameters only without body dimension measurements are considered poor estimators for body composition [91,100]. Diaz *et al.* [101] concluded that in FM and FFM prediction, resistance and capacitance measurements contribute by 0%–20%. In contrast, the percentages increase to 11%–53% after height inclusion, and 22%–68% after inclusion of Ht^2^/R ratio.

Ward *et al.* [102] presented a validation study to predict BCM and ECF as a portion of TBW without measuring height and using BIA device, the Soft Tissue Analyzer STA^TM^ (Akern Sri, Florence, Italy) with a correlation coefficient referenced to the total body potassium counting method is equal to 0.91, 0.82 and 0.89, and a standard estimation error equal of 5.6 kg, 6.3 kg and 1.3 kg for FFM, BCM and ECF, respectively.

### 4.2. Gender

Variations in body composition between male and female were proven in several studies [103]. In body composition prediction, methods based on bioimpedance analysis, and most equations tend to include gender as one of the main determining factors for body compartment assessment [13,86,87].

FFM or lean mass studies show that males have greater FFM than females with different ranges. Kyle *et al.* [81] state that mean FFM for male is 8.9 kg and 6.2 kg for female and fat mass index FMI increases based on age, in females from 5.6 to 9.4 and from 3.7 to 7.4 in males. In a recent study [104] on 1649 healthy children-adults (6–18 years) and 925 adult-elders (19–92 years) using BIA and DXA it was concluded that for all age ranges, males have less fat mass and more fat free mass than females.

TBW averaged 73.2% of fat free mass in the healthy population; however several studies show that males have less TBW than females [11]. Sun *et al.* [86], stated that in a mixed ethnic groups prediction equation, TBW volume for males start from 1.2 L compared with 3.75 L for females. Jaffrin *et al.* [31] state that determined TBW resistivity (ρ) is on average 104.3 ± 7.9 Ω·cm for men and 100.5 ± 7.8 Ω·cm for women. The values are smaller in men are due to their larger limb cross section.

Due to the different body composition between males and females, gender considerations have a strong impact in estimating body compartments.

### 4.3. Age

Aging is defined as a multi-factor changing in the physical and biological activities of the human body that leads to differences in body composition among age groups. When the human body becomes older it leads to a gradual increase in fat mass and spontaneous decrease in lean mass. Fat free mass to fat mass ratio increases gradually in response to increase of age, and a noticeable increment in average weight is seen among the elder population compared with adults associated with increment in fat mass [81]. In some studies [58], the general body composition prediction equations were unsatisfactory in elderly men over 75 years of age, especially in TBW estimation.

Several studies were conducted using the BIA method on children [68,105] adults [13], and elders [106,107]. In children, the BIA method using the Deurenberg equation [87], underestimates body fat as determined by DXA. It however achieved a better correlation than the skin fold method [108]. Muscle mass loss among the elderly reduces the fat free mass at a certain age, followed by decreases in total body water and bone mass [109]. Marja *et al.* [107] reported that in 75-year-old Swedes, average fat free mass index was 15.6 and 18.3; and body fat index was 11.0 and 8.6 for women and men, respectively, compared to the DXA method.

### 4.4. Ethnic Groups

Body composition varies among different races and ethnic groups due to the environment, nutrition factors, culture and anthropometric measurements that include body conformation [110]. There is also difference in limb length [111], body structure [112], body size [89] and that lead to variation in body fat percentages among different ethnic groups which may lead to prediction errors (3%) [111].

The majority of bioimpedance measurement studies have been done on Caucasian subjects [5], Kotler *et al.* [113] and Sun *et al.* [86] have included African American and Hispanic subjects in their studies. Kim *et al.* assessed the segmental lean mass among Koreans [106], Schulz *et al.* assessed the fat free mass among Germans and compared it to the American and Swiss population [114]. Siváková *et al.* studied the clinical applications of BIVA on Slovaks [115]. Nigam *et al.* had performed a comparative study among two different Indian races [116], whereas Saragat *et al.* obtained specific BIVA reference values for the Italian healthy elderly population in order to construct the specific tolerance ellipses to be used for reference purposes for assessing body composition in gerontological practice and for epidemiological purposes [117]. Validation of bioimpedance measurements among different ethnicities is thus needed due to differences in body composition among certain populations.

### 4.5. Measurements Protocols and Posture

Simplicity and the economic acceptance of bioimpedance analysis method for body composition estimation have increased the need to unify the protocols and procedures of bioimpedance measurements in order to retrieve robust data.

For the foot to ankle measurement method, bioimpedance measurements performed in a supine position with abduction of the upper limbs to 30 degrees and lower limbs to 45 degrees for 5 to 10 min. studies show that when the posture changes from a standing to a supine body position, the ECV decreased in the arms by 2.51% and legs by 3.02%, but increased in the trunk by 3.2% [118]. Fasting for at least 8 hours and bladder voiding before measurements are recommended as consumption of food and beverages may decrease impedance by 4–15 O over a 2–4 h period after meals and that causes an error (<3%) [84,119,120]. Body anthropometric measurements should be retrieved prior of the test and for scale or foot to foot bioimpedance analyzer weight retrieved automatically [1].

Electrodes should be placed on the pre-cleaned metacarpal and metatarsal phalangeal joints with a distance in between of at least 5 cm without skin lesions at the location of the electrodes. In some studies skin temperature should be counted [84,120]. Subjects under test should not perform any exercise activities before measurements that could lead to errors in assessed resistance and reactance equal to 3% and 8% respectively [121]. Roos *et al.* concluded that the error in total body water prediction range from 1 to 1.5 L figured out after laying at rest for one hour [122].

### 4.6. Electrode Shape and Measurement Error

In bioimpedance analysis, the geometrical structure of electrode has a strong impact on elementary data retrieved during the measurement process. In bioimpedance analysis electrodes are defined as isoelectric materials with a negligible voltage drop along the connectors. The minimum numbers of electrodes required to perform the bioimpedance measurements are two, one for current injection with the assumption of zero potential difference and the other for collecting the voltage drop with a negligible current flow and is more affected by position.

The tetrapolar electrode approach become widely used for whole bioimpedance measurements because of the uniformity of current distribution compared to monopolar electrodes [6], and the usage of more than two potential collecting electrodes or octapolar electrode method were used for segmental bioimpedance studies to assess compartments in different body segments [73].

Ag-AgCl electrodes are now used in most bioimpedance measurements because it has a well-defined DC potential with electrolyte gel to minimize the gap impedance between skin and electrodes. Circular and rectangular electrode shapes with a contact area greater than 4 cm^2^ are the most commonly used shapes [1].

Buendía *et al.* investigated the impact of electrode discrepancy on BIS measurements and concluded that mismatched potential electrode causes 4% overestimated measurements in resistance at zero and infinite frequency because of an imbalanced electrical field distribution [123]. Shiffman [124] addressed the artifacts caused by inaccurate distance between electrodes in four electrode measurement methods performed on a 17.5 cm segment of the thigh area. That study reported that the values of resistance and reactance were four times larger when the current injecting electrodes were placed 2.5 cm from the sensing electrodes. Scharfetter *et al.* stated that capacitance between different body segments and earth, and capacitance between the signal ground of the device and earth cause a significant false dispersion in the measured impedance spectra at frequencies >500 kHz [125].

Errors in bioimpedance measurements are caused by many factors such as motion, miss-positioning, connector length and fabrication errors. Moreover, the diversity of the commercially available bioimpedance analyzers cause a wide range of fluctuations in measurements between the devices. Thus the calibration of the components inside a bioimpedance analyzer such as signal generator, sensing apparatus, scales of weight and height and electrical interference should be conducted to ensure the reliability of the bioimpedance analyzers [1].

## 5. Applications of Bioimpedance Analysis in Clinical Status Monitoring and Diagnosis of Diseases

Bioimpedance analysis in healthcare practice contributes to the estimation of body compartments to assess the regular change in nutrition status in in-patients and to monitor nutritional risk in out-patients [126]. Most of the body composition assessment methods like BMI techniques, skin fold method and underwater weight measurements is used to estimate fat mass and fat free mass, however bioimpedance analysis can estimate FM and FFM in addition to total and particular body fluids which is very helpful for disease prognosis [127]. The National Health and Nutrition Examination Survey program in United States included bioimpedance analysis in the third NHANES program between 1999 and 2004 to assess the health and nutritional status of adults and children because of a general frustration with the dependability of the skin fold thickness method to estimate FM and FFM, especially in subjects with higher amount of segmented fat [128].

Observation of body compartment fluctuations like fat free mass, fat mass and total body water from normal limits are considered as key factors to be used in bioimpedance analysis in healthcare applications. Abnormal loss in lean body mass and unbalanced shifts in body fluids are the most measured parameters to be used to assess the healthiness of the human body. Analysis of bioimpedance parameters has bern used in several studies to estimate and analyze the changes in disorders of different kind of diseases.

Norman *et al.* [70] stated that phase angle is an essential predictor of clinical status. Pichler *et al.* [91] stated that estimation of body fluids using BIS was slightly better than anthropometric methods among healthy and diseased.

Table 1 contains some of the applications of bioimpedance analysis in disease diagnosis that are organized according to the organ systems of human body, diseases or abnormalities diagnosed based on bioimpedance parameters, and comments on how these factors are applied to determine the health condition. Bioimpedance analysis is a common method used for estimating body composition among healthy and diseased subjects in research and clinical trials. This review has focused on the theoretical and the fundamentals of bioimpedance analysis. Thus it may have some limitations, where possible important studies on the applications of bioimpedance analysis in diagnostic of diseases and the related shifts in bioimpedance parameters may have been missed.

## 6. Conclusions

Increasing demands for accurate, cost effective and non-invasive systems for clinical status monitoring and diagnosis of diseases in healthcare, has accelerated the research endeavors to provide new methods and technologies to evaluate the health condition of human body. Body composition assessment tools has been considered a promising approach for the quantitative measurement of tissues characteristic over time, in addition to direct relativity between fluctuations in body composition equivalences and survival rate, clinical condition, illness and quality of life. Bioimpedance analysis is a growing method for body compartments estimation in nutrition studies, sport medicine and evaluation of hydration rate, fat mass and fat free mass between healthy and diseased populations. Fat mass, fat free mass including skeletal muscle mass, bone minerals, and total body water, which is composed of intercellular fluid and extracellular fluid, are compartments that can be predicted and analyzed using suitable bioimpedance measurements techniques, procedures and population, age, ethnic groups or disease-dedicated bioimpedance analysis equations. Further studies are needed to evaluate the correlations between variations in bioimpedance parameters, especially in ECF and ICF, and the deviation from health to disease.

## Figures and Tables

**Table 1.** Applications of bioimpedance analysis in clinical status monitoring and diagnosis of diseases.

Organ Systems	Diseases	BIA Parameters	Remarks	Authors
Pulmonary system	Lung cancer, stages IIIB and IV	R and X_c_ (BIVA)	Reactance components decrease in patients (phase angle <4.5). *Clinical Study.*	Toso *et al.*, 2000 [129]

	Pulmonary edema monitoring	R (SFBIA)	Mean resistivity for left and right lung (1205 ± 163, 1200 ± 165 Ω·cm) and system reproducibility (2%). *Research Study.*	Zlochiver *et al.*, 2007 [130]

Cardio-vascular system	Fluid accumulation after cardiac surgery.	Ht^2^/Z (MFBIA)	Significant increase in segmental trunk bioimpedance after surgery due to fluid accumulation. *Clinical Study.*	Bracco *et al.*, 1998 [131]

Circulatory system	Volaemic status and hyponatraemia	TBW (SFBIA)	In elderly hyponatraemic patients, TBW assessment using BIA method was correlated with dilution of deuterium oxide (R = 0.68). *Clinical Study.*	Hoyle *et al.*, 2011 [132]

	Hydration status and hyponatraemia in elderly	TBW (SFBIA)	Assessment of hydration status in elderly hyponatraemic patients using BIA method was more accurate than clinical procedures (Cohen's kappa coefficient = 0.52). *Clinical Study.*	Cumming *et al.*, 2014 [133]

Renal system	Chronic hemodialysis	ECF (BIS)	ECF to weight ratio of hypertensive patient's increase from that of normal patients (24.29 ± 3.56% *vs.* 21.50 ± 2.38). *Clinical Study.*	Chen *et al.*, 2002 [134]

	Dry weight in kidney failure.	ECF (BIS)	ECF/Wt is 0.239 and 0.214 L/kg for male and female healthy subjects. *Clinical Study.*	Chamney *et al.*, 2002 [135]

	Hydration states monitoring in hemodialysis patients	Calf-BIS (BIS)	Normalized resistivity (μ = ρ /BMI) increased from 17.9 ± 3 to 19.1 ± 2.3 × 10^−2^ Ω^3^·Kg^−1^, and weight was reduced from 78.3 ± 28 to 77.1 ± 27 kg in Post-dialysis. *Research Study.*	Zhu *et al.*, 2007 [136], 2008 [137]

	Dry weight assessment hemodialysis patients	Calf-BIS (BIS)	Dry weight assessed by cBIS underestimate left ventricular mass and blood pressure while antihypertensive medication remains unchanged. *Clinical Study.*	Seibert *et al.*, 2013 [138]

	Body fluids estimation in hemodialysis patients	ECF, ICF and TBW (BIS)	Correlation between proposed equation corrected for BMI and the references (mean ± SD) was −0.4 ± 1.4 L for ECF, 0.2 ± 2.0 L for ICF and −0.2 ± 2.3 L for TBW. *Clinical Study.*	Moissl *et al.*, 2006 [97]

	Dry weight assessment HD patients	R and X_c_ (BIVA)	BIVA method shows significant different in vectors in post dialysed patients. *Clinical Study.*	Atilano *et al.*, 2012 [139]

Neural system	Alzheimer's disease	R and X_c_ (BIVA)	BCM decreased in patients for men, T^2^ (Hotelling's statistic) = 12.8 and for women, T^2^ = 34.9. *Clinical Study*.	Buffa *et al.*, 2010 [140]

	Anorexia nervosa (eating disorder)	FM, FFM, TBW and ECF (BIS)	The BCM to Ht^2^ ratio was found to be significantly changed between diseased and controls subjects. *Clinical Study.*	Moreno *et al.*, 2008 [141]

	Anorexia nervosa (eating disorder)	R and X_c_ (BIVA)	Gradually increasing in BCM and decreasing in ECF during treatments. *Clinical Study.*	Haas *et al.*, 2012 [69]

Muscular system	Body composition changes monitoring during exercise training	FFM and FM (MFBIA)	BIA method underestimates FM (−3.42 kg) and overestimated FFM (3.18 kg); and undetected small shift in body composition due to exercise training. *Clinical Study.*	Sillanpää *et al.*, 2013 [142]

Immunology system	Comparison between SFBIA and MFBIA in HIV patients	ECF and TBW (BIS)	Insignificant differences in TBW and ECF estimation using SFBIA, MFBIA and BIS methods. *Clinical Study.*	Paton *et al.*, 1998 [143]
	Dengue haemorrhagic fever estimation in children	ECF and ICF (BIS)	(ECF/ICF) increase with increasing dengue virus infections severity in children. *Clinical Study.*	Libraty *et al.*, 2002 [144]

	Cancer patients	TBW (SFBIA)	Change in TBW using BIA method (Ht^2^/R_50_) correlate with deuterium dilution in underweight and normal-weight cancer patients (R^2^ = 0.43 and SEE = 1.22 L). *Clinical Study.*	Simons *et al.*, 1999 [145]

	Early diagnosis and risk analysis of dengue	R, C, φ and Xc (SFBIA)	Reactance variations among dengue patients during defervescence of feverintervalis an indicator for classifying risk category in the DHF patients. *Clinical Study.*	Ibrahim *et al.*, [146]

Other diseases	Critically ill subjects	FM, TBW and ECF (BIS)	Body composition using BIS method show slightly more significant in estimation of FM, TBW and ECF among healthy and diseased subjects. *Clinical Study.*	Pichler *et al.*, 2013 [91]
	Gastrointestinal disease	R, Xc, Fc, FFM, TBW, ECF and ICF (BIS)	In critically diseased subjects, Fc and ECF increased, Xc decreased, and TBW and ICF remain the same. *Clinical Study.*	Cox-Reijven *et al.*, 2003 [67]

## Notes

**Author Contributions** All authors contributed extensively to the work presented in this paper.

## References

[1] Kyle U.G., Bosaeus I., De Lorenzo A.D., Deurenberg P., Elia M., Manuel Gómez J., Lilienthal Heitmann B., Kent-Smith L., Melchior J.-C., Pirlich M. (2004). Bioelectrical impedance analysis—Part ii: Utilization in clinical practice. Clin. Nutr..

[2] Thomasset A. (1962). Bio-electrical properties of tissue impedance measurements. Lyon Med..

[3] Nyboer J., Thomas C. (1970). Electrical Impedance Plethysmograph.

[4] Hoffer E.C., Meador C.K., Simpson D.C. (1969). Correlation of whole-body impedance with total body water volume. J. Appl. Physiol..

[5] Kyle U.G., Bosaeus I., De Lorenzo A.D., Deurenberg P., Elia M., Gómez J.M., Heitmann B.L., Kent-Smith L., Melchior J.-C., Pirlich M. (2004). Bioelectrical impedance analysis—Part i: Review of principles and methods. Clin. Nutr..

[6] Martinsen O.G., Grimnes S. (2011). Bioimpedance and Bioelectricity Basics.

[7] Mialich M.S., Sicchieri J.M.F., Junior A.A.J. (2014). Analysis of body composition: A critical review of the use of bioelectrical impedance analysis. Int. J. Clin. Nutr..

[8] Lukaski H. (2013). Evolution of bioimpedance: A circuitous journey from estimation of physiological function to assessment of body composition and a return to clinical research. Eur. J. Clin. Nutr..

[9] Kasap S.O. (1997). Principles of Electrical Engineering Materials and Devices.

[10] De Lorenzo A., Andreoli A., Matthie J., Withers P. (1997). Predicting body cell mass with bioimpedance by using theoretical methods: A technological review. J. Appl. Physiol..

[11] Genton L., Hans D., Kyle U.G., Pichard C. (2002). Dual-energy x-ray absorptiometry and body composition: Differences between devices and comparison with reference methods. Nutrition.

[12] Thomas B., Ward L., Cornish B. (1998). Bioimpedance spectrometry in the determination of body water compartments: Accuracy and clinical significance. Appl. Radiat. Isot..

[13] Kyle U.G., Genton L., Karsegard L., Slosman D.O., Pichard C. (2001). Single prediction equation for bioelectrical impedance analysis in adults aged 20–94 years. Nutrition.

[14] Gudivaka R., Schoeller D., Kushner R., Bolt M. (1999). Single-and multifrequency models for bioelectrical impedance analysis of body water compartments. J. Appl. Physiol..

[15] Chertow G.M., Lazarus J.M., Lew N.L., Ma L., Lowrie E.G. (1997). Development of a population-specific regression equation to estimate total body water in hemodialysis patients. Kidney Int..

[16] Ward L.C., Dyer J.M., Byrne N.M., Sharpe K.K., Hills A.P. (2007). Validation of a three-frequency bioimpedance spectroscopic method for body composition analysis. Nutrition.

[17] Lukaski H.C., Bolonchuk W.W., Hall C.B., Siders W.A. (1986). Validation of tetrapolar bioelectrical impedance method to assess human body composition. J. Appl. Physiol..

[18] Hanai T. (1961). Electrical properties of emulsions. Kolloid-Zeitschrift.

[19] Olde R.M., Deurenberg P., Jansen R., Van't Hof M., Hoefnagels W. (1997). Validation of multi-frequency bioelectrical impedance analysis in detecting changes in fluid balance of geriatric patients. J. Am. Geriatr. Soc..

[20] Woodrow G., Oldroyd B., Turney J., Davies P., Day J., Smith M. (1996). Measurement of total body water by bioelectrical impedance in chronic renal failure. Eur. J. Clin. Nutr..

[21] Jaffrin M.Y., Morel H. (2008). Body fluid volumes measurements by impedance: A review of bioimpedance spectroscopy (bis) and bioimpedance analysis (bia) methods. Med. Eng. Phys..

[22] Simpson J., Lobo D., Anderson J., Macdonald I., Perkins A., Neal K., Allison S., Rowlands B. (2001). Body water compartment measurements: A comparison of bioelectrical impedance analysis with tritium and sodium bromide dilution techniques. Clin. Nutr..

[23] Pierson R., Wang J., Colt E., Neumann P. (1982). Body composition measurements in normal man: The potassium, sodium, sulfate and tritium spaces in 58 adults. J. Chronic Dis..

[24] Patel R.V., Peterson E.L., Silverman N., Zarowitz B.J. (1996). Estimation of total body and extracellular water in post-coronary artery bypass graft surgical patients using single and multiple frequency bioimpedance. Crit. Care Med..

[25] Thomasset A. (1963). Bio-electrical properties of tissues. Lyon Med..

[26] Cole K.S., Cole R.H. (1941). Dispersion and absorption in dielectrics i. Alternating current characteristics. J. Chem. Phys..

[27] Cornish B., Ward L., Thomas B., Jebb S., Elia M. (1996). Evaluation of multiple frequency bioelectrical impedance and cole-cole analysis for the assessment of body water volumes in healthy humans. Eur. J. Clin. Nutr..

[28] Pastan S., Gassensmith C. (1992). Total body water measured by bioelectrical impedance in patients after hemodialysis: Comparison with urea kinetics. ASAIO J..

[29] Scanferla F., Landini S., Fracasso A., Morachiello P., Righetto F., Toffoletto P., Bazzato G. (1990). On-line bioelectric impedance during haemodialysis: Monitoring of body fluids and cell membrane status. Nephrol. Dial Transplant..

[30] Ellis K.J., Wong W.W. (1998). Human hydrometry: Comparison of multifrequency bioelectrical impedance with 2H_2_O and bromine dilution. J. Appl. Physiol..

[31] Jaffrin M.Y., Fenech M., Moreno M.V., Kieffer R. (2006). Total body water measurement by a modification of the bioimpedance spectroscopy method. Med. Biol. Eng. Comput..

[32] Matthie J.R. (2005). Second generation mixture theory equation for estimating intracellular water using bioimpedance spectroscopy. J. Appl. Physiol..

[33] Matthie J., Zarowitz B., De Lorenzo A., Andreoli A., Katzarski K., Pan G., Withers P. (1998). Analytic assessment of the various bioimpedance methods used to estimate body water. J. Appl.Physiol..

[34] Baarends E., Van Marken Lichtenbelt W., Wouters E., Schols A. (1998). Body-water compartments measured by bio-electrical impedance spectroscopy in patients with chronic obstructive pulmonary disease. Clin. Nutr..

[35] Cox-Reijven P., Soeters P. (2000). Validation of bio-impedance spectroscopy: Effects of degree of obesity and ways of calculating volumes from measured resistance values. Int. J. Obes..

[36] Earthman C.P., Matthie J.R., Reid P.M., Harper I.T., Ravussin E., Howell W.H. (2000). A comparison of bioimpedance methods for detection of body cell mass change in hiv infection. J. Appl. Physiol..

[37] Ward L., Elia M., Cornish B. (1998). Potential errors in the application of mixture theory to multifrequency bioelectrical impedance analysis. Physiol. Meas..

[38] Hannan W., Cowen S., Plester C., Fearon K., DeBeau A. (1995). Comparison of bieimpedance spectroscopy and mu it if requency biei m pedance analysis for the assessment of extracellular and total body water in surgical patients. Clin. Sci..

[39] Deurenberg P., Andreoli A., De Lorenzo A. (1996). Multi-frequency bioelectrical impedance: A comparison between the cole-cole modelling and hanai equations with the classical impedance index approach. Ann. Hum. Biol..

[40] Scharfetter H., Monif M., Laszlo Z., Lambauer T., Hutten H., Hinghofer-Szalkay H. (1997). Effect of postural changes on the reliability of volume estimations from bioimpedance spectroscopy data. Kidney Int..

[41] Ayllon D., Seoane F., Gil-Pita R. Cole equation and parameter estimation from electrical bioimpedance spectroscopy measurements-a comparative study.

[42] Ward L.C., Essex T., Cornish B.H. (2006). Determination of cole parameters in multiple frequency bioelectrical impedance analysis using only the measurement of impedances. Physiol. Meas..

[43] Cole K.S. (1968). Membranes, Ions, and Impulses: A Chapter of Classical Biophysics.

[44] Xie X., Kolthoff N., Bärenholt O., Nielsen S. (1999). Validation of a leg-to-leg bioimpedance analysis system in assessing body composition in postmenopausal women. Int. J. Obes..

[45] Jebb S.A., Cole T.J., Doman D., Murgatroyd P.R., Prentice A.M. (2000). Evaluation of the novel tanita body-fat analyser to measure body composition by comparison with a four-compartment model. Br. J. Nutr..

[46] Utter A.C., Nieman D.C., Ward A.N., Butterworth D.E. (1999). Use of the leg-to-leg bioelectrical impedance method in assessing body-composition change in obese women. Am. J. Clin. Nutr..

[47] Deurenberg P., Deurenberg‐Yap M. (2002). Validation of skinfold thickness and hand‐held impedance measurements for estimation of body fat percentage among singaporean chinese, malay and indian subjects. Asia Pac. J. Clin. Nutr..

[48] Ghosh S., Meister D., Cowen S., Hannan J.W., Ferguson A. (1997). Body composition at the bedside. Eur. J. Gastroenterol. Hepatol..

[49] Buchholz A.C., Bartok C., Schoeller D.A. (2004). The validity of bioelectrical impedance models in clinical populations. Nutr. Clin. Pract..

[50] Nuñez C., Gallagher D., Visser M., Pi-Sunyer F.X., Wang Z., Heymsfield S.B. (1997). Bioimpedance analysis: Evaluation of leg-to-leg system based on pressure contact footpad electrodes. Med. Sci. Sports Exerc..

[51] Tanaka N.I., Miyatani M., Masuo Y., Fukunaga T., Kanehisa H. (2007). Applicability of a segmental bioelectrical impedance analysis for predicting the whole body skeletal muscle volume. J. Appl. Physiol..

[52] Baumgartner R.N., Chumlea W.C., Roche A.F. (1988). Bioelectric impedance phase angle and body composition. Am. J. Clin. Nutr..

[53] Thomas B., Cornish B., Ward L., Patterson M. (1998). A comparison of segmental and wrist-to-ankle methodologies of bioimpedance analysis. Appl. Radiat. Isot..

[54] Thomas B., Cornish B., Pattemore M., Jacobs M., Ward L. (2003). A comparison of the whole-body and segmental methodologies of bioimpedance analysis. Acta Diabetol..

[55] Earthman C., Traughber D., Dobratz J., Howell W. (2007). Bioimpedance spectroscopy for clinical assessment of fluid distribution and body cell mass. Nutr. Clin. Pract..

[56] Moon J. (2013). Body composition in athletes and sports nutrition: An examination of the bioimpedance analysis technique. Eur. J. Clin. Nutr..

[57] Fogelholm M., van Marken L.W. (1997). Comparison of body composition methods: A literature analysis. Eur. J. Clin. Nutr..

[58] Fuller N., Sawyer M., Laskey M., Paxton P., Elia M. (1996). Prediction of body composition in elderly men over 75 years of age. Ann. Hum. Biol..

[59] Chumlea W., Schubert C., Sun S., Demerath E., Towne B., Siervogel R. (2007). A review of body water status and the effects of age and body fatness in children and adults. J. Nutr. Health Aging.

[60] Coppini L.Z., Waitzberg D.L., Campos A.C.L. (2005). Limitations and validation of bioelectrical impedance analysis in morbidly obese patients. Curr. Opin Clin. Nutr. Metab. Care.

[61] Rutkove S.B., Aaron R., Shiffman C.A. (2002). Localized bioimpedance analysis in the evaluation of neuromuscular disease. Muscle Nerve.

[62] Jaffrin M.Y. (2009). Body composition determination by bioimpedance: An update. Curr. Opin. Clin. Nutr. Metab. Care.

[63] Thomas B., Cornish B., Ward L. (1992). Bioelectrical impedance analysis for measurement of body fluid volumes: A review. J. Clin. Eng..

[64] Piccoli A., Piazza P., Noventa D., Pillon L., Zaccaria M. (1996). A new method for monitoring hydration at high altitude by bioimpedance analysis. Med. Sci. Sports Exerc..

[65] Piccoli A., Rossi B., Pillon L., Bucciante G. (1994). A new method for monitoring body fluid variation by bioimpedance analysis: The rxc graph. Kidney Int..

[66] Piccoli A., Pillon L., Dumler F. (2002). Impedance vector distribution by sex, race, body mass index, and age in the united states: Standard reference intervals as bivariate scores. Nutrition.

[67] Cox-Reijven P.L., van Kreel B., Soeters P.B. (2003). Bioelectrical impedance measurements in patients with gastrointestinal disease: Validation of the spectrum approach and a comparison of different methods for screening for nutritional depletion. Am. J. Clin. Nutr..

[68] Azevedo Z.M.A., Moore D.C.B.C., de Matos F.A.A., Fonseca V.M., Peixoto M.V.M., Gaspar-Elsas M.I.C., Santinoni E., dos Anjos L.A., Ramos E.G. (2013). Bioelectrical impedance parameters in critically ill children: Importance of reactance and resistance. Clin. Nutr..

[69] Haas V., Riedl A., Hofmann T., Nischan A., Burghardt R., Boschmann M., Klapp B. (2012). Bioimpedance and bioimpedance vector analysis in patients with anorexia nervosa. Eur. Eat. Disord. Rev..

[70] Norman K., Stobäus N., Pirlich M., Bosy-Westphal A. (2012). Bioelectrical phase angle and impedance vector analysis–clinical relevance and applicability of impedance parameters. Clin. Nutr..

[71] Ward L.C., Heitmann B.L. (2000). Re: “Electrical maturation trajectory of human tissues identified by bioelectrical impedance vector analysis”. Nutrition.

[72] Marini E., Sergi G., Succa V., Saragat B., Sarti S., Coin A., Manzato E., Buffa R. (2013). Efficacy of specific bioelectrical impedance vector analysis (biva) for assessing body composition in the elderly. J. Nutr. Health Aging.

[73] Bracco D., Thiébaud D., Chioléro R.L., Landry M., Burckhardt P., Schutz Y. (1996). Segmental body composition assessed by bioelectrical impedance analysis and dexa in humans. J. Appl. Physiol..

[74] Yanovski S.Z., Hubbard V.S., Heymsfield S.B., Lukaski H.C. (1996). Bioelectrical impedance analysis in body composition measurement: National institutes of health technology assessment conference statement. Am. J. Clin. Nutr..

[75] Sanchez B., Vandersteen G., Bragos R., Schoukens J. (2012). Basics of broadband impedance spectroscopy measurements using periodic excitations. Meas. Sci. Technol..

[76] Sanchez B., Bandarenka A.S., Vandersteen G., Schoukens J., Bragos R. (2013). Novel approach of processing electrical bioimpedance data using differential impedance analysis. Med. Eng. Phys..

[77] Sanchez B., Schoukens J., Bragos R., Vandersteen G. (2011). Novel estimation of the electrical bioimpedance using the local polynomial method. Application to *in vivo* real-time myocardium tissue impedance characterization during the cardiac cycle. IEEE Trans. Biomed. Eng..

[78] Sanchez B., Vandersteen G., Bragos R., Schoukens J. (2011). Optimal multisine excitation design for broadband electrical impedance spectroscopy. Med. Eng. Phys..

[79] Sanchez B., Rojas C.R., Vandersteen G., Bragos R., Schoukens J. (2012). On the calculation of the d-optimal multisine excitation power spectrum for broadband impedance spectroscopy measurements. Med. Eng. Phys..

[80] Sanchez B., Louarroudi E., Jorge E., Cinca J., Bragos R., Pintelon R. (2013). A new measuring and identification approach for time-varying bioimpedance using multisine electrical impedance spectroscopy. Physiol. Meas..

[81] Kyle U.G., Genton L., Slosman D.O., Pichard C. (2001). Fat-free and fat mass percentiles in 5225 healthy subjects aged 15 to 98 years. Nutrition.

[82] Roubenoff R., Dallal G.E., Wilson P. (1995). Predicting body fatness: The body mass index *vs.* estimation by bioelectrical impedance. Am. J. Public Health.

[83] Kyle U.G., Pichard C. (2000). Dynamic assessment of fat-free mass during catabolism and recovery. Curr. Opin. Clin. Nutr. Metab. Care.

[84] Heitmann B. (1994). Impedance: A valid method in assessment of body composition?. Eur. J. Clin. Nutr..

[85] Kyle U.G., Schutz Y., Dupertuis Y.M., Pichard C. (2003). Body composition interpretation: Contributions of the fat-free mass index and the body fat mass index. Nutrition.

[86] Sun S.S., Chumlea W.C., Heymsfield S.B., Lukaski H.C., Schoeller D., Friedl K., Kuczmarski R.J., Flegal K.M., Johnson C.L., Hubbard V.S. (2003). Development of bioelectrical impedance analysis prediction equations for body composition with the use of a multicomponent model for use in epidemiologic surveys. Am. J. Clin. Nutr..

[87] Deurenberg P., Van der Kooy K., Leenen R., Weststrate J., Seidell J. (1991). Sex and age specific prediction formulas for estimating body composition from bioelectrical impedance: A cross-validation study. Int. J. Obes..

[88] Pichard C., Kyle U.G., Bracco D., Slosman D.O., Morabia A., Schutz Y. (2000). Reference values of fat-free and fat masses by bioelectrical impedance analysis in 3393 healthy subjects. Nutrition.

[89] Deurenberg P., Weststrate J.A., Seidell J.C. (1991). Body mass index as a measure of body fatness: Age-and sex-specific prediction formulas. Br. J. Nutr..

[90] Heitmann B.L. (1990). Evaluation of body fat estimated from body mass index, skinfolds and impedance. A comparative study. Eur. J. Clin. Nutr..

[91] Pichler G.P., Amouzadeh-Ghadikolai O., Leis A., Skrabal F. (2013). A critical analysis of whole body bioimpedance spectroscopy (BIS) for the estimation of body compartments in health and disease. Med. Eng. Phys..

[92] Sargent J.A., Gotch F.A. (1989). Principles and biophysics of dialysis. Replacement of Renal Function by Dialysis.

[93] Lukaski H.C., Johnson P.E., Bolonchuk W., Lykken G. (1985). Assessment of fat-free mass using bioelectrical impedance measurements of the human body. Am. J. Clin. Nutr..

[94] Sergi G., Bussolotto M., Perini P., Calliari I., Giantin V., Ceccon A., Scanferla F., Bressan M., Moschini G., Enzi G. (1994). Accuracy of bioelectrical impedance analysis in estimation of extracellular space in healthy subjects and in fluid retention states. Ann. Nutr. Metab..

[95] Deurenberg P., Tagliabue A., Schouten F.J. (1995). Multi-frequency impedance for the prediction of extracellular water and total body water. Br. J. Nutr..

[96] Schloerb P.R., Friis-Hansen B.J., Edelman I.S., Solomon A., Moore F.D. (1950). The measurement of total body water in the human subject by deuterium oxide dilution: With a consideration of the dynamics of deuterium distribution 1. J. Clin. Investig..

[97] Moissl U.M., Wabel P., Chamney P.W., Bosaeus I., Levin N.W., Bosy-Westphal A., Korth O., Müller M.J., Ellegård L., Malmros V. (2006). Body fluid volume determination via body composition spectroscopy in health and disease. Physiol. Meas..

[98] Wagner D.R., Heyward V.H. (1999). Techniques of body composition assessment: A review of laboratory and field methods. Res. Q. Exerc. Sport.

[99] Serrano M.D.M., de Espinosa M.G.-M., Zamorano E.M. (2012). Relationship between physical measures of anthropometry and bioimpedance measures. Handbook of Anthropometry.

[100] Jaffrin M.Y., Bousbiat S., Dongmo E. (2011). A comparison between two methods for measuring limb resistances with wrist and ankle electrodes. Med. Eng. Phys..

[101] Diaz E., Villar J., Immink M., Gonzales T. (1989). Bioimpedance or anthropometry?. Eur. J. Clin. Nutr..

[102] Ward L., Heitmann B. (2001). Assessment of body composition by bioelectrical impedance analysis without the need for measurement of height. Clin. Nutr..

[103] Mridha S. (2010). A comparative study on body composition of male and female national level sub-junior volleyball players. Br. J. Sports Med..

[104] Kirchengast S. (2010). Gender differences in body composition from childhood to old age: An evolutionary point of view. J. Life Sci..

[105] Fomon S.J., Haschke F., Ziegler E.E., Nelson S.E. (1982). Body composition of reference children from birth to age 10 years. Am. J. Clin. Nutr..

[106] Kim J.H., Choi S.H., Lim S., Kim K.W., Lim J.Y., Cho N.H., Park K.S., Jang H.C. (2014). Assessment of appendicular skeletal muscle mass by bioimpedance in older community-dwelling korean adults. Arch Gerontol. Geriatr..

[107] Tengvall M., Ellegård L., Malmros V., Bosaeus N., Lissner L., Bosaeus I. (2009). Body composition in the elderly: Reference values and bioelectrical impedance spectroscopy to predict total body skeletal muscle mass. Clin. Nutr..

[108] Eisenmann J.C., Heelan K.A., Welk G.J. (2004). Assessing body composition among 3‐ to 8‐year‐old children: Anthropometry, bia, and dxa. Obes. Res..

[109] Buffa R., Floris G.U., Putzu P.F., Marini E. (2011). Body composition variations in ageing. Coll. Antropol..

[110] Deurenberg P., Deurenberg-Yap M., Schouten F. (2002). Validity of total and segmental impedance measurements for prediction of body composition across ethnic population groups. Eur. J. Clin. Nutr..

[111] Deurenberg P., Deurenberg-Yap M. (2003). Validity of body composition methods across ethnic population groups. Acta Diabetol..

[112] Deurenberg P., Wolde-Gebriel Z., Schouten F. (1995). Validity of predicted total body water and extracellular water using multifrequency bioelectrical impedance in an ethiopian population. Ann. Nutr. Metab..

[113] Kotler D.P., Burastero S., Wang J., Pierson R. (1996). Prediction of body cell mass, fat-free mass, and total body water with bioelectrical impedance analysis: Effects of race, sex, and disease. Am. J. Clin. Nutr..

[114] Schulz H., Teske D., Penven D., Tomczak J. (2006). Fat-free mass from two prediction equations for bioelectrical impedance analysis in a large german population compared with values in swiss and american adults: Reasons for a biadata project. Nutrition.

[115] Siváková D., Vondrová D., Valkovič P., Cvíčelová M., Danková Z., Luptáková L. (2013). Bioelectrical impedance vector analysis (biva) in slovak population: Application in a clinical sample. Cent. Eur. J. Biol..

[116] Nigam P., Misra A., Colles S.L. (2013). Comparison of dexa-derived body fat measurement to two race-specific bioelectrical impedance equations in healthy indians. Diabetes Metab. Syndr..

[117] Saragat B., Buffa R., Mereu E., De Rui M., Coin A., Sergi G., Marini E. (2014). Specific bioelectrical impedance vector reference values for assessing body composition in the italian elderly. Exp. Gerontol..

[118] Zhu F., Schneditz D., Wang E., Levin N.W. (1998). Dynamics of segmental extracellular volumes during changes in body position by bioimpedance analysis. J. Appl. Physiol..

[119] Schols A., Dingemans A., Soeters P., Wouters E. (1990). Within-day variation of bioelectrical resistance measurements in patients with chronic obstructive pulmonary disease. Clin. Nutr..

[120] Kushner R.F., Gudivaka R., Schoeller D.A. (1996). Clinical characteristics influencing bioelectrical impedance analysis measurements. Am. J. Clin. Nutr..

[121] Liang M., Norris S. (1993). Effects of skin blood flow and temperature on bioelectric impedance after exercise. Med. Sci. Sports Exerc..

[122] Roos A., Westendorp R., Frölich M., Meinders A. (1992). Tetrapolar body impedance is influenced by body posture and plasma sodium concentration. Eur. J. Clin. Nutr..

[123] Buendía R., Bogónez-Franco P., Nescolarde L., Seoane F. (2012). Influence of electrode mismatch on cole parameter estimation from total right side electrical bioimpedance spectroscopy measurements. Med. Eng. Phys..

[124] Shiffman C. (2013). Adverse effects of near current-electrode placement in non-invasive bio-impedance measurements. Physiol. Meas..

[125] Scharfetter H., Hartinger P., Hinghofer-Szalkay H., Hutten H. (1998). A model of artefacts produced by stray capacitance during whole body or segmental bioimpedance spectroscopy. Physiol. Meas..

[126] Kondrup J., Allison S., Elia M., Vellas B., Plauth M. (2003). Espen guidelines for nutrition screening 2002. Clin. Nutr..

[127] Thibault R., Genton L., Pichard C. (2012). Body composition: Why, when and for who?. Clin. Nutr..

[128] Kuczmarski R.J. (1996). Bioelectrical impedance analysis measurements as part of a national nutrition survey. Am. J. Clin. Nutr..

[129] Toso S., Piccoli A., Gusella M., Menon D., Bononi A., Crepaldi G., Ferrazzi E. (2000). Altered tissue electric properties in lung cancer patients as detected by bioelectric impedance vector analysis. Nutrition.

[130] Zlochiver S., Arad M., Radai M., Barak-Shinar D., Krief H., Engelman T., Ben-Yehuda R., Adunsky A., Abboud S. (2007). A portable bio-impedance system for monitoring lung resistivity. Med. Eng. Phys..

[131] Bracco D., Revelly J.-P., Berger M.M., Chiolero R.L. (1998). Bedside determination of fluid accumulation after cardiac surgery using segmental bioelectrical impedance. Crit. Care Med..

[132] Hoyle G., Chua M., Soiza R. (2011). Volaemic assessment of the elderly hyponatraemic patient: Reliability of clinical assessment and validation of bioelectrical impedance analysis. QJM.

[133] Cumming K., Hoyle G., Hutchison J., Soiza R.L. Bioelectrical impedance analysis is more accurate than clinical examination in determining the volaemic status of elderly patients with fragility fracture and hyponatraemia. J. Nutr. Health Aging.

[134] Chen Y.-C., Chen H.-H., Yeh J.-C., Chen S.-Y. (2002). Adjusting dry weight by extracellular volume and body composition in hemodialysis patients. Nephron.

[135] Chamney P.W., Krämer M., Rode C., Kleinekofort W., Wizemann V. (2002). A new technique for establishing dry weight in hemodialysis patients via whole body bioimpedance. Kidney Int..

[136] Zhu F., Kuhlman M., Kotanko P., Handelman G., Leonard E., Levin N. A device for monitoring hydration state in hemodialysis patients using a calf bioimpedance technique.

[137] Zhu F., Kuhlmann M., Kotanko P., Seibert E., Leonard E., Levin N. (2008). A method for the estimation of hydration state during hemodialysis using a calf bioimpedance technique. Physiol. Meas..

[138] Seibert E., Mueller S.G., Fries P., Pattmoeller J., Kuss O., Heine G.H., Girndt M., Schneider G., Kotanko P., Zhu F. (2013). Calf bioimpedance spectroscopy for determination of dry weight in hemodialysis patients: Effects on hypertension and left ventricular hypertrophy. Kidney Blood Press. Res..

[139] Atilano X., Luis Miguel J., Martínez J., Sánchez R., Selgas R. (2012). Bioimpedance vector analysis as a tool for determination and adjustment of dry weight in hemodialysis patients. Kidney Res. Clin. Prac..

[140] Buffa R., Mereu R., Putzu P., Floris G., Marini E. (2010). Bioelectrical impedance vector analysis detects low body cell mass and dehydration in patients with alzheimer's disease. J. Nutr. Health Aging.

[141] Moreno M.V., Djeddi D.-D., Jaffrin M.Y. (2008). Assessment of body composition in adolescent subjects with anorexia nervosa by bioimpedance. Med. Eng. Phys..

[142] Sillanpää E., Häkkinen A., Häkkinen K. (2013). Body composition changes by dxa, bia and skinfolds during exercise training in women. Eur. J. Appl. Physiol..

[143] Paton N.I., Elia M., Jennings G., Ward L.C., Griffin G.E. (1998). Bioelectrical impedance analysis in human immunodeficiency virus-infected patients: Comparison of single frequency with multifrequency, spectroscopy, and other novel approaches. Nutrition.

[144] Libraty D.H., Endy T.P., Kalayanarooj S., Chansiriwongs W., Nisalak A., Green S., Ennis F.A., Rothman A.L. (2002). Assessment of body fluid compartment volumes by multifrequency bioelectrical impedance spectroscopy in children with dengue. Trans. R. Soc. Trop. Med. Hyg..

[145] Simons J., Schols A., Westerterp K., Ten Velde G., Woliters E. (1999). Bioelectrical impedance analysis to assess changes intotal body water in patients with cancer. Clin. Nutr..

[146] Ibrahim F., Taib M.N., Abas W.A.B.W., Guan C.C., Sulaiman S. (2005). A novel approach to classify risk in dengue hemorrhagic fever (dhf) using bioelectrical impedance analysis (bia). IEEE Trans. Instrum. Meas..

